# Association Between Cytomegalovirus Viremia Clearance and Post-Solid Organ Transplant Mortality in Patients With Refractory Cytomegalovirus Infection: SOLSTICE Post Hoc Analysis

**DOI:** 10.3389/ti.2025.15331

**Published:** 2025-11-26

**Authors:** Nassim Kamar, Robin K. Avery, Tien Bo, Joan Gu, Deepali Kumar, Oliver Witzke

**Affiliations:** 1 Toulouse Rangueil University Hospital, INFINITY-Inserm U1291-CNRS U5051, University Paul Sabatier, Toulouse, France; 2 School of Medicine, Johns Hopkins University, Baltimore, MD, United States; 3 Takeda Development Center Americas, Inc., Cambridge, MA, United States; 4 Ajmera Transplant Centre, University Health Network, Toronto, ON, Canada; 5 Department of Infectious Diseases, West German Centre of Infectious Diseases, University Medicine Essen, University Duisburg-Essen, Essen, Germany

**Keywords:** SOT (solid organ transplant), survival, anti-CMV therapy, maribavir, antiviral

## Abstract

Cytomegalovirus (CMV) infection following solid organ transplant (SOT) is associated with increased mortality risk. In the phase 3 SOLSTICE study (NCT02931539), more transplant recipients achieved CMV clearance after 8 weeks with maribavir than investigator-assigned therapy (IAT). In SOLSTICE, SOT recipients with refractory CMV infection were randomized 2:1 to receive maribavir or IAT for 8 weeks. This *post hoc* analysis assessed the impact of CMV clearance at Week 8 on mortality at Week 20. Patients who achieved CMV clearance at Week 8 were categorized as responders, and patients without CMV clearance, or who received maribavir rescue or alternative treatment, were categorized as nonresponders. All-cause mortality was assessed at Week 20 for responders and nonresponders using the Kaplan-Meier method with log-rank test. The analysis included 211 SOT recipients: 97 responders and 114 nonresponders. Week 20 all-cause mortality was significantly higher in nonresponders than responders (p = 0.0024). No deaths were reported in the responder group, and 10 deaths were reported in the nonresponder group (3 receiving IAT, 7 receiving maribavir). Median (range) time from treatment start to death was 30.5 (3–123) days. This analysis is consistent with other studies showing an increased risk of mortality with post-SOT CMV infection.

## Graphical Abstract

**Figure:**
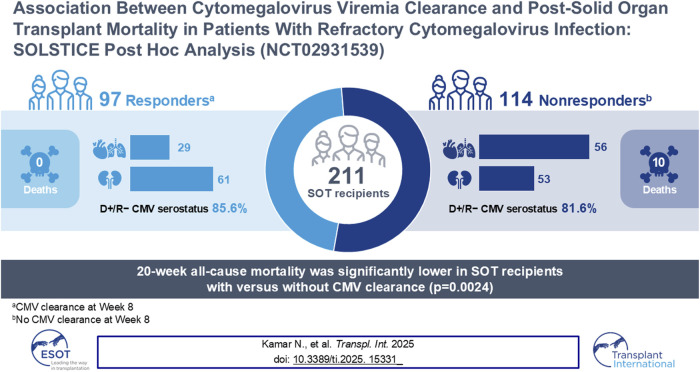

## Introduction

Transplantation is a life-saving procedure with mean survival after solid organ transplant (SOT) estimated at 20–27 years for kidney and liver transplants, 15–16 years for heart transplants, and 9 years for lung transplants [1]. The most common causes of death in SOT recipients are cancer, graft failure, infection, and cardiovascular disease [2]. Studies of mortality in SOT recipients have identified several risk factors, including older age, male sex, acute rejection, low platelet count, and post-transplant infection [3–7].

Cytomegalovirus (CMV) is one of the most common pathogens associated with infection in SOT recipients [7, 8]. Data from a systematic review estimates the incidence of CMV infection between 5.2% and 76.4% dependent on the transplanted organ and treatment strategy [9]. Importantly, post-SOT CMV infection is associated with an increased risk of graft loss and mortality [7, 8, 10].

Established antiviral agents for management of post-SOT CMV are ganciclovir and its oral prodrug valganciclovir, despite the high incidence of myelosuppression with these agents [11–14]. Alternatively, in (val)ganciclovir resistant or refractory cases, intravenous foscarnet can be used. However, foscarnet is associated with nephrotoxicity and electrolyte depletion, thereby compromising patient safety [12, 14, 15].

First approved in 2021, maribavir is an oral treatment for post-transplant CMV infection/disease that is refractory (with or without resistance) to prior antiviral drugs or intolerant to treatment (in certain countries) [16–18]. The phase 3 SOLSTICE study compared maribavir with investigator-assigned therapy (IAT; valganciclovir/ganciclovir, foscarnet, or cidofovir) in patients with refractory post-transplant CMV infection, with or without genotypically documented resistance [19]. The results showed that significantly more patients receiving maribavir achieved CMV clearance at the end of Week 8 compared with IAT (55.7% vs. 23.9%; adjusted difference [95% confidence interval (CI)]: 32.8% [22.80–42.74]; p < 0.001) and achieved CMV clearance and symptom control at the end of Week 8 that was maintained through Week 16 (18.7% vs. 10.3%; adjusted difference [95% CI]: 9.5% [2.02–16.88]; p = 0.01). Moreover, in the SOT population, a higher proportion of maribavir-treated patients achieved these endpoints across transplant organ types [20]. Maribavir-treated patients also had less neutropenia and less acute kidney injury compared with valganciclovir/ganciclovir- and foscarnet-treated patients, respectively [19]. Here we present data from a *post hoc* analysis of the SOLSTICE study designed to assess the impact of CMV viremia clearance at Week 8 on Week 20 mortality among SOT recipients with refractory CMV infection.

## Patients and Methods

Full details of the SOLSTICE study (ClinicalTrials.gov: NCT02931539) have been published previously [19]. In brief, SOLSTICE was a phase 3, randomized, open-label, multicenter study. Transplant recipients (SOT or hematopoietic cell transplant) were aged ≥12 years with CMV DNA ≥910 IU/mL at screening and were refractory (with or without resistance) to their most recent CMV treatment. Patients were randomized 2:1 to receive maribavir 400 mg twice daily or investigator’s choice of monotherapy or combination therapy with ganciclovir/valganciclovir, foscarnet, or cidofovir for 8 weeks, with an additional 12 weeks of follow-up. The primary endpoint was confirmed CMV viremia clearance at the end of Week 8 [19].

In the present *post hoc* analysis, patients were categorized based on whether they achieved the primary endpoint. Responders were defined as patients with plasma CMV DNA below the lower limit of quantification (137 IU/mL) in 2 consecutive post-baseline samples, separated by ≥5 days at Week 8. Nonresponders were defined as patients who did not achieve CMV clearance at Week 8 (including those with missing virologic data) or who received maribavir rescue or alternative treatment before the end of Week 8.

All-cause mortality at Week 20 was plotted for responders and nonresponders (to maribavir or IAT) using the Kaplan-Meier method with log-rank test. Recipient characteristics were summarized using descriptive statistics and compared between responder and nonresponder groups using the chi-square test. Multivariate analyses were conducted using Cox proportional hazards model for Week 20 mortality. Analyses were conducted using SAS® 9.4.

## Results

The 211 SOT recipients from the SOLSTICE study were included in the analysis, of whom 97 were categorized as responders and 114 as nonresponders (Supplementary Figure S1). Baseline characteristics were broadly similar; the only statistically significant difference in baseline characteristics between responders and nonresponders was transplanted organ (p = 0.0170) (Table 1). Most patients were kidney or lung recipients, and the majority had donor-positive/recipient-negative (D+/R−) CMV serostatus. More recipients in the nonresponder group had high CMV DNA levels (≥91,000 IU/mL) than responders (n = 21, 18.4% vs. n = 13, 13.4%).

**TABLE 1:** Baseline characteristic of solid organ transplant recipients in the SOLSTICE study.

Characteristic	Responders (n = 97)	Nonresponders (n = 114)	*p-*value
Age, median (range), years	56 (25–77)	55 (19–79)	0.5701^a^
Sex, n (%)			0.3195
Male	70 (72.2)	75 (65.8)	
Female	27 (27.8)	39 (34.2)	
Transplant type, n (%)			0.0170
Kidney	61 (62.9)	53 (46.5)	
Heart and/or lung	29 (29.9)	56 (49.1)	
Other	7 (7.2)	5 (4.4)	
Graft status at baseline, n (%)			0.1236
Functioning	85 (87.6)	103 (90.4)	
Functioning with complications	12 (12.4)	8 (7.0)	
Other	0	3 (2.6)	
Retransplant			0.0681
Yes	8 (8.2)	19 (16.7)	
No	89 (91.8)	95 (83.3)	
CMV serostatus, n (%)			
D+/R−	83 (85.6)	93 (81.6)	
D+/R+	7 (7.2)	12 (10.5)	
D−/R−	6 (6.2)	4 (3.5)	
D−/R+	1 (1.0)	3 (2.6)	
Missing	0	2 (1.8)	
CMV DNA levels at randomization, n (%)			0.3278
Low (<9,100 IU/mL)	45 (46.4)	42 (36.8)	
Intermediate (≥9,100 to <91,000 IU/mL)	39 (40.2)	51 (44.7)	
High (≥91,000 IU/mL)	13 (13.4)	21 (18.4)	
Cardiovascular disease			0.8529
Yes	7 (7.2)	9 (7.9)	
No	90 (92.8)	105 (92.1)	
Diabetes			0.2250
Yes	2 (2.1)	6 (5.3)	
No	95 (97.9)	108 (94.7)	
Anti-lymphocyte use			0.7220
Yes	32 (33.0)	35 (30.7)	
No	65 (67.0)	79 (69.3)	

^a^ Analysis conducted for <65 vs. ≥65 years.

CMV, cytomegalovirus; D, donor; R, recipient.

**FIGURE 1:**
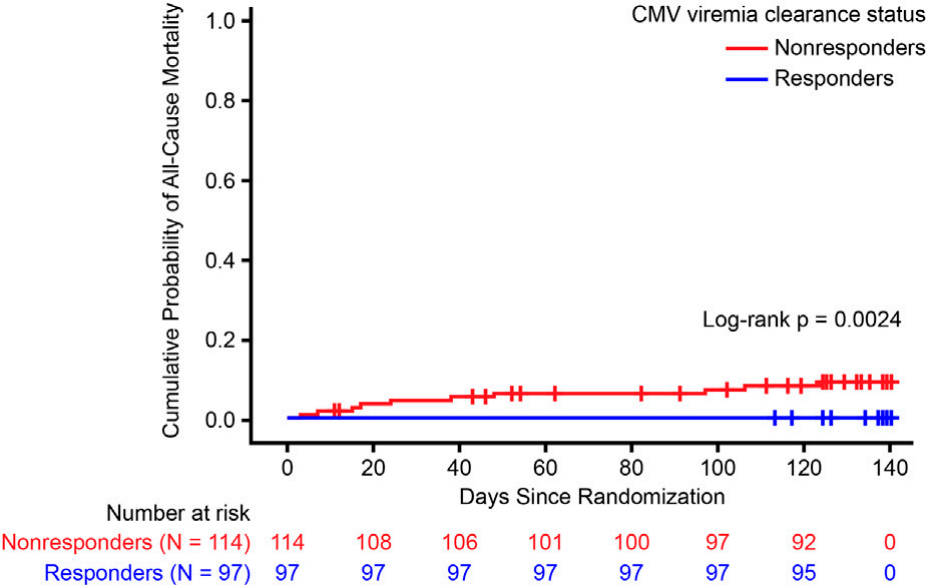
Cumulative probability of all-cause mortality at Week 20 in solid organ transplant recipients categorized by viremia clearance at Week 8. CMV, cytomegalovirus.

**FIGURE 2:**
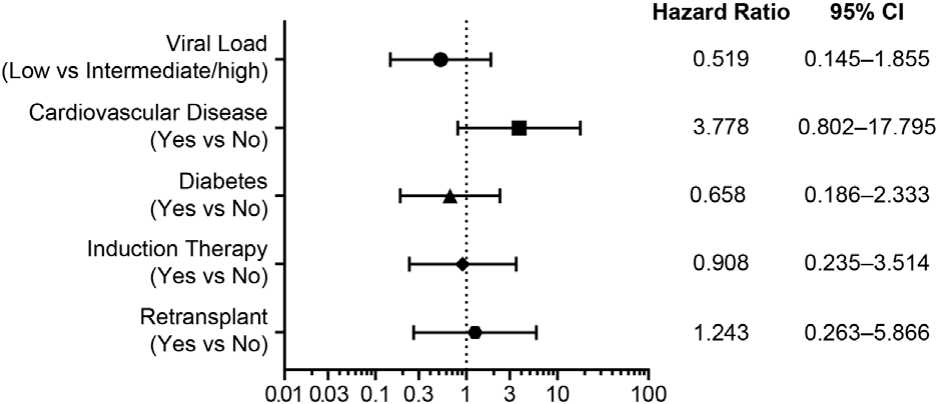
Cox regression analysis showing hazard ratios and 95% confidence intervals (CIs) for risk factors associated with mortality in transplant recipients with CMV. Transplant type was excluded from this analysis due to the small sample size within each category.

In SOT recipients, 20-week all-cause mortality was significantly lower in responders than in nonresponders (p = 0.0024) (Figure 1). By Week 20, no deaths were reported in responders compared with 3 deaths in nonresponders who received IAT and 7 deaths in nonresponders who received maribavir (Table 2). Cox regression analysis did not show any significant risk factors for death among baseline characteristics for viral load, cardiovascular disease, diabetes, induction therapy, or retransplant (Figure 2). Median (range) CMV viral load at the last measurement was 2,001.5 IU/mL (68.5–67,539.0) in nonresponders who died compared with 68.5 IU/mL (68.5–621,920.0) and 68.5 IU/mL (68.5–38,733.0) in nonresponders and responders, respectively, who survived (Table 3).

**TABLE 2:** Mortality outcomes at Week 20 in SOT recipients categorized by viremia clearance at Week 8.

Cytomegalovirus viremia clearance status	Participants n/N	Events n (%)^a^	Time to event, median (range), days
Nonresponders			
Investigator-assigned treatment	51/69	3 (5.9)	38.0 (17.0–106.0)
Maribavir	63/142	7 (11.1)	24.0 (3.0–124.0)
Responders			
Investigator-assigned treatment	18/69	0	–
Maribavir	79/142	0	–

SOT, solid organ transplant.

^a^ Denominator is number of participants in each response category within each treatment group (n).

N, total number of SOT, recipients in each treatment group; n, number of participants in each response category within each treatment group.

**TABLE 3:** CMV viral load (IU/mL) at last measurement.

Treatment	Responders		Nonresponders	
	Died	Survived	Died	Survived
Maribavir, n	0	79	7	56
Median (range)	-	68.5 (68.5–3,071.0)	2,730.0 (68.5–67,539.0)	68.5 (68.5–27,871.0)
IAT, n	0	18	3	48
Median (range)	-	68.5 (68.5–38,733.0)	1,882.0 (1,132.0–2,121.0)	68.5 (68.5–621,920.0)
Total, n	0	97	10	104
Median (range)	-	68.5 (68.5–38,733.0)	2,001.5 (68.5–67,539.0)	68.5 (68.5–621,920.0)

One of the deaths was considered related to maribavir by the investigator due to a drug interaction with posaconazole causing sudden cardiac death by arrhythmia. Other causes of death included respiratory failure (n = 3) and pulmonary embolism (n = 2). CMV infection contributed to cause of death in 4 participants. The characteristics of recipients who died and causes of death are shown in Table 4. Of the 10 recipients who died, 6 were male, and the median (range) age was 60.5 (28–77) years. Two recipients had received more than 1 transplant before study entry. Eight of the 10 recipients had cardiovascular comorbidities. No patients had a recent history of organ rejection, and all had functioning transplants at baseline. CMV serostatus was D+/R− in 8 of 10 (80%) patients who died, similar to the percentage of D+/R− in the cohort as a whole. The group who died included 1 patient who had received rescue therapy with maribavir after foscarnet. The median (range) time from SOT to treatment start was 253 (72–549) days, from treatment start to treatment end was 15 (2–58) days, from treatment start to death was 30.5 (3–123) days, and from treatment end to death was 9 (1–72) days. Only 2 of the patients who died completed the treatment course; of the 8 who discontinued, 5 were due to death.

**TABLE 4:** Patient characteristics of nonresponders who died.

SOT type	CMV serostatus, D/R	CV comorbidities	CMV syndrome	Viral load category^a^	Lymphocyte count at baseline^b^	Study drug	Length of Tx, days	Time from SOT to Tx start, days	Time from Tx start to death, days	Time from Tx end to death, days	Cause of death
Kidney	+/−	Y	N	I	0.1	GCV	8	279	38	30	CMV-related pneumonia
Lung	+/−	Y	N	L	1.1	FSC	16	165	17	1	Respiratory failure
Kidney	+/+	Y	N	H	0.2	FSC	34	253	106	72	Unknown^c^
Heart	−/+	Y	Y	L	0.8^d^	MBV	47	116	48	1	Cardiac arrest
Lung	+/−	N	Y	L	0.7	MBV	2	123	3	1	Deep vein thrombosis with probable progression to pulmonary embolus
Lung	+/−	Y	N	I	1.7	MBV	58	549	94	36	Respiratory failure
Kidney	+/−	N	Y	I	0.6	MBV	4	319	7	3	Drug-drug interaction with an outcome of sudden death^e^
Kidney	+/−	Y	N	I	0.1	MBV	8	253	23	15	Respiratory failure/CMV syndrome
Heart	+/−	Y	N	L	0.6	MBV	14	434	15	1	Massive pulmonary embolism
Heart	+/−	Y	N	I	0.9^f^	MBV	56	72	123	67	Extensive venous thrombosis

CMV, cytomegalovirus; CV, cardiovascular; D/R, donor/recipient; FSC, foscarnet; GCV, ganciclovir; H, high; I, intermediate; L, low; MBV, maribavir; N, no; NA, not applicable; QTc, corrected QT, interval; Tx, treatment; Y, yes.

^a^ Baseline plasma CMV DNA, viral load was defined as low, <9,100 IU/mL; intermediate, ≥9,100 to <91,000 IU/mL; or high, ≥91,000 IU/mL.

^b^ Baseline measurement taken 1 day from the first dose of treatment.

^c^ Foscarnet-treated patient qualified for maribavir rescue (inadequate response and toxicity) [19] and developed CMV, encephalitis 2 days after maribavir initiation, patient died (cause unknown) 49 days after last dose of maribavir.

^d^ Measurement taken 15 days from first dose of treatment.

^e^ The investigator interpreted this event as sudden cardiac death due to arrhythmia and reported it as possible drug interaction. The patient had also received voriconazole, posaconazole, and domperidone. In studies of healthy adults, doses of maribavir up to 1,200 mg were not shown to prolong QTc [21].

^f^ Measurement taken −1 day from the first dose of treatment.

## Discussion

This *post hoc* analysis of data from the phase 3, randomized SOLSTICE study showed that SOT recipients with refractory CMV infection who achieved CMV clearance at Week 8 had a significantly reduced probability of 20-week all-cause mortality than those without CMV clearance.

The present study adds to the body of evidence confirming that post-transplant CMV infection is a known risk factor for mortality in SOT recipients [4, 7, 22]. For example, a retrospective analysis of 1,454 renal transplant recipients showed that CMV disease was associated with a 2.5-fold increase in risk of death (p < 0.001) [4]. Similarly, a retrospective study of 88 lung transplant recipients showed that CMV disease was associated with a 4.2-fold increase in mortality (p = 0.002), whereas CMV infection and disease were associated with a 3.8-fold increase in mortality (p = 0.001) [7]. In a longitudinal study of CMV infection and viral load in 2,510 sequential kidney transplant recipients, CMV infection was associated with a significantly increased risk of death (p = 0.0056) [22].

Cox regression analysis did not identify any significant risk factors for mortality among the following baseline characteristics: viral load, cardiovascular disease, diabetes, induction therapy, and retransplant. As expected, median CMV viral load at latest measurement was higher in patients who died than in those who survived.

In the present analysis, 9 of the 10 recipients who died received transplants from CMV-positive donors. It is known that CMV-positive donor status is associated with a higher risk of CMV infection [22] and mortality [23, 24]. A high proportion of the recipients who died in this analysis also had cardiovascular comorbidities, which are known risk factors for mortality following SOT [25, 26]. In this analysis, only 2 recipient deaths were caused by cardiac arrest, one of which was due to drug interactions. In this case, the investigator assigned the sudden cardiac death as possibly related to maribavir. However, doses of maribavir up to 1,200 mg in healthy adults have been shown not to prolong QTc [21]. The patient had also received posaconazole, voriconazole, and domperidone. Domperidone is associated with an increased risk of sudden cardiac death and arrhythmia and should not be taken with voriconazole [27, 28]. Half of the deaths in this analysis were due to respiratory failure and pulmonary embolism. Three patients receiving maribavir experienced thrombosis or pulmonary embolism; however, none of these were deemed as related to maribavir. Baseline characteristics of nonresponders included higher proportions of lung or heart transplants and intermediate/high viral loads. Chi-square analysis showed a significant difference between responders and nonresponders for kidney, heart or lung, and other transplanted organs.

The association between CMV clearance and mortality in the present analysis does not necessarily mean that CMV clearance was the sole reason for reduced mortality. All-cause mortality is likely to be driven by multiple factors, and CMV clearance at 8 weeks of treatment may be a marker of the patient’s general health, disease severity, or immune status. However, it does appear that failure to clear CMV identifies a group with an increased risk of death potentially due to other causes not directly related to CMV. Therefore, the nonresponder group may merit close follow-up and preventive care from the standpoint of cardiovascular issues and venous thromboembolism, as well as ongoing anti-CMV treatment.

Data from this *post hoc* analysis should be interpreted with caution, as mortality was not the primary endpoint of the SOLSTICE study. Moreover, the number of patients included in the analysis may not provide adequate power and may not reflect a thorough assessment of the underlying cause of mortality [29]. The potential for selection bias should also be taken into consideration.

In conclusion, achievement of viremia clearance at Week 8 was associated with a significantly reduced probability of mortality at Week 20. While many factors likely affect mortality in transplant recipients with refractory or resistant CMV infection, these findings suggest that achieving and maintaining CMV clearance in this complex patient population is a contributing factor to survival. Application of these findings to clinical practice is worthy of further study.

## Data Availability

The datasets presented in this article are not readily available. The datasets, including the redacted study protocol, redacted statistical analysis plan, and individual participants data supporting the results reported in this article, will be made available within three months from initial request, to researchers who provide a methodologically sound proposal. The data will be provided after its deidentification, in compliance with applicable privacy laws, data protection, and requirements for consent and anonymization. Requests to access the datasets should be directed to TB, tien.bo@takeda.com.
