# Supplementary material for: Association Between Cytomegalovirus Viremia Clearance and Post-Solid Organ Transplant Mortality in Patients With Refractory Cytomegalovirus Infection: SOLSTICE Post Hoc Analysis
Source: Transpl Int. 2025 Nov 26;38:15331. doi: 10.3389/ti.2025.15331 (PMC12689443; doi:10.3389/ti.2025.15331)
Supplement: Supplementary file 1 [file Image1.pdf]

SUPPLEMENTARY MATERIAL

Figure S1 | Patient disposition

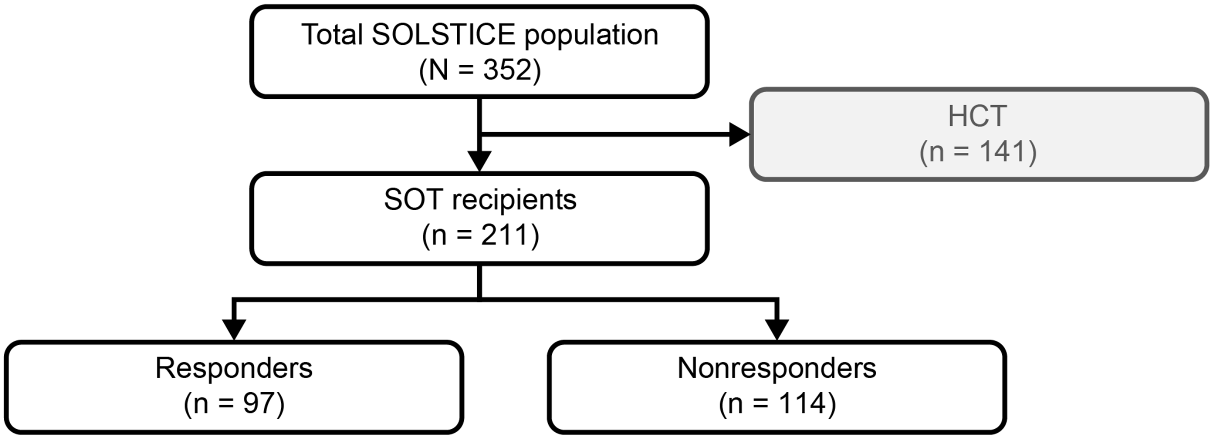

HCT, hematopoietic cell transplant; SOT, solid organ transplant.
